# Supplementary material for: An exponential build-up in seismic energy suggests a months-long nucleation of slow slip in Cascadia
Source: Nat Commun. 2020 Aug 18;11:4139. doi: 10.1038/s41467-020-17754-9 (PMC7435189; doi:10.1038/s41467-020-17754-9)
Supplement: Supplementary file 1 — Supplementary Information [file 41467_2020_17754_MOESM1_ESM.pdf]

Supplementary Information for An Exponential Build-up in Seismic Energy Suggests a Months-Long Nucleation of Slow Slip **Events** in Cascadia

Claudia Hulbert[1,2], Bertrand Rouet-Leduc[2], R. Jolivet[1,3], Paul A. Johnson[2]

[1] Laboratoire de géologie, Département de Géosciences, Ecole Normale Supérieure, PSL Université, CNRS UMR 8538, Paris, France

[2]Los Alamos National Laboratory, Geophysics Group, Los Alamos, New Mexico, USA

[3]Institut Universitaire de France, 1 rue Descartes, 75005 Paris.

*C. Hulbert (email: claudia.hulbert@ens.fr)

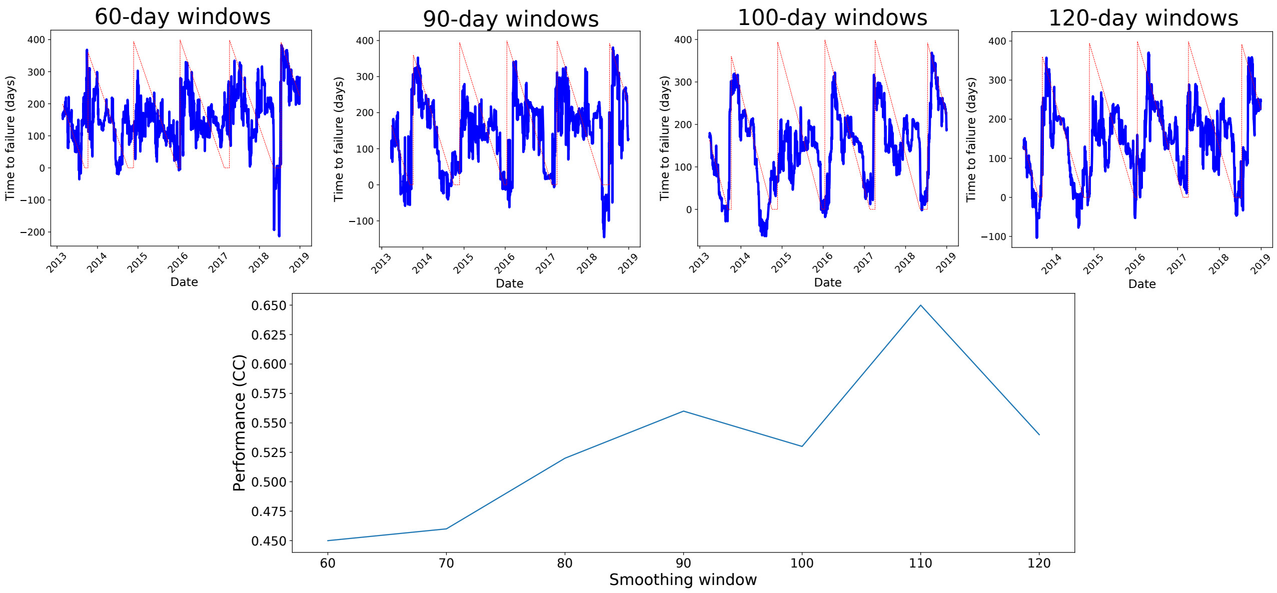

Supplementary Figure 1. Impact of the size of the window used to parse the seismic data on the performance of the ML algorithm, for the best seismic station (B001). Our results are robust to changes in the window length used to parse the seismic data. However, we find that seismic data is noisy, even for the borehole stations analyzed, and that large windows are required to smooth out the noise. The ML estimations using two months of seismic data are clearly noisy, but the results become rapidly smoother as the window size increases. Figure 3 of the main text shows results with a window of 3 months, the smallest window that leads to relatively smooth results. Larger windows can lead to smoother estimations and better performance. The bottom plot shows the impact on the performance in testing for a broader range of window sizes.

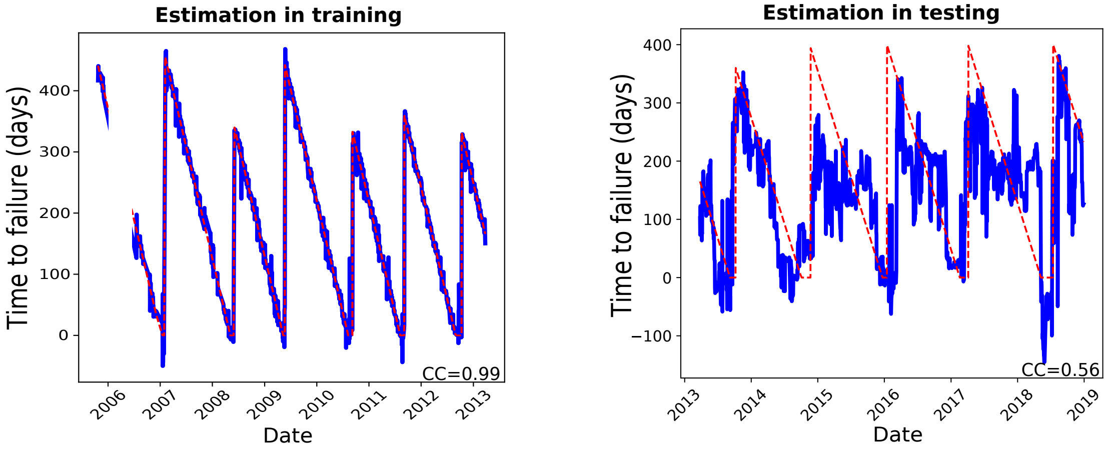

Supplementary Figure 2. Performance in training (left) and testing (right) for the model trained on 3-month windows (same as the Figure 3A of the paper). Gaps correspond to missing data. Note that the algorithm could have selected higher levels of L1 and L2 penalization while performing the Bayesian optimization of the hyperparameters, but chose to rely on intermediate values during cross-validation instead.

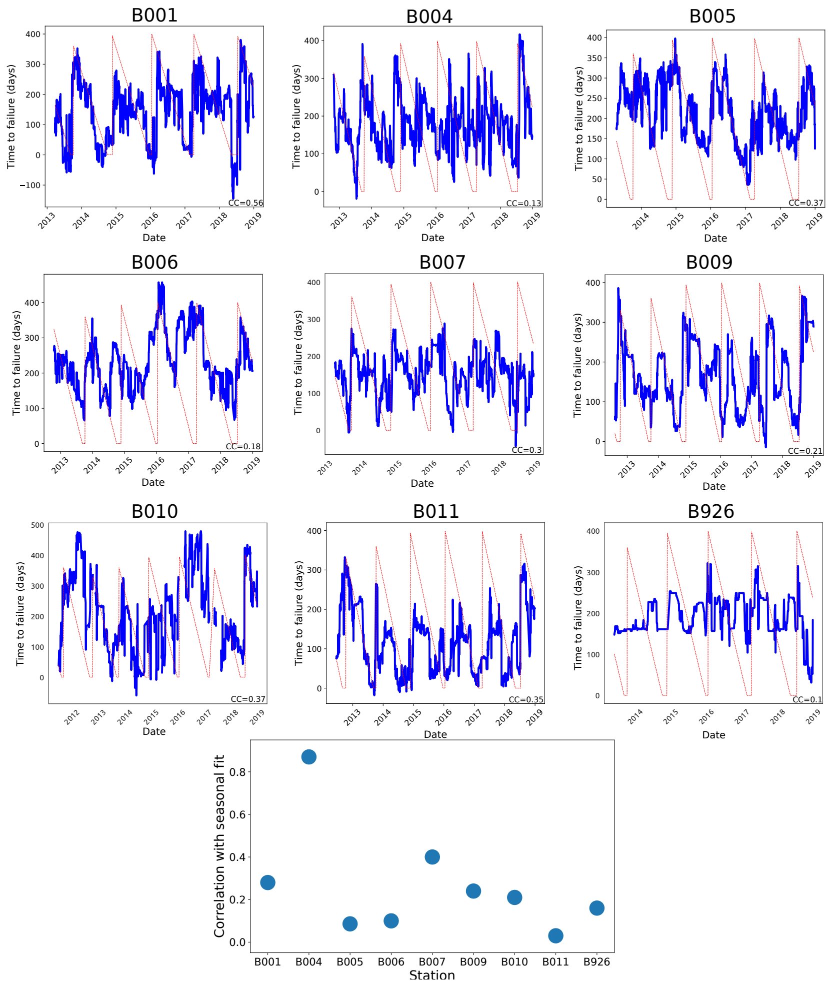

Supplementary Figure 3. Comparison of model performance in testing using the different seismic stations. We build a different model for each of the seismic stations. Stacking station features or providing multiple stations as input to the algorithm does not seem to improve the results. We find that a specific seismic station (B001) is consistently better for the exercise, regardless of the time window used for the analysis. This station is **right above the center of** an active tremor area, which may explain why it performs better (see Figure 1 of the main text). It is characterized by a strong increase in seismic power close to failure, and a small level of noise (Figure 5 of the main text). The stations B009 and B011 also show structure when compared to slip timing, although they do not perform as well as B001 (these **stations** are located very close to an airport, which may be the origin of noise detrimental to our analysis). Surprisingly, the almost co-located station B010 does not perform well at all; this could be explained by its very close proximity to the ocean (about 10m). Site effects and proximity to anthropogenic and natural noise sources therefore appear to matter significantly for the analysis, and only stations with a particularly low level of noise can be used successfully. The bottom plot shows the correlation between the feature shown in the paper (60-40 inter-quantile range, in the 8-9Hz band) with a seasonal sinusoidal. Except for B004, the stations do not seem heavily corrupted by seasonal signals.

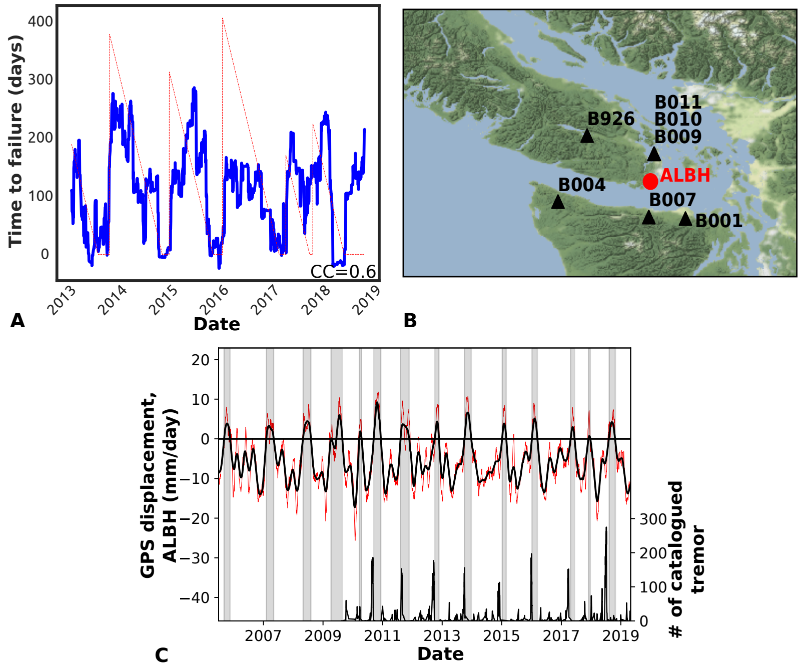

Supplementary Figure 4. (A) Performance of the ML prediction in testing (blue) to estimate time to failure (red) when using GPS to identify slow slip failure times (see Supplementary Methods). The map (B) shows the location of the ALBH GPS station. (C) GPS displacement rate at site ALBH as a function of time, projected in the southwest direction. Positive values (grey shades) indicate displacement rate toward the southwest (i.e. indicative of a slow slip event) while negative values indicate displacement rate toward the northeast (i.e. indicative of locking along the megathrust).

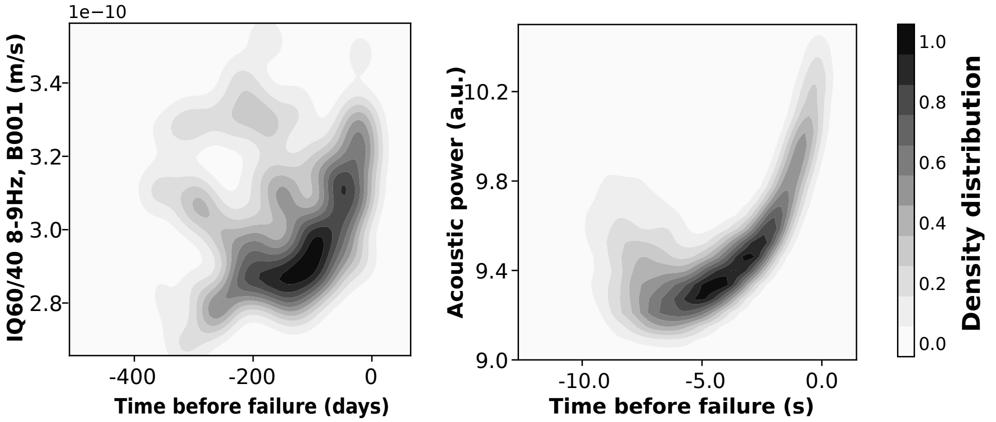

Supplementary Figure 5. Evolution of seismic noise preceding failure, with slip timings identified by GPS in Cascadia. Left: Cascadia slow slip events. Right: laboratory earthquakes. As mentioned in the main text **and above**, identifying slip timings with GPS may introduce contamination from ongoing slow slip events located elsewhere on the subduction zone. The increased performance of our model when we use GPS instead of PNSN Tremor Logs for slip timing is therefore likely due to this contamination. The exponential behaviour of seismic noise preceding failure is clearer with the GPS slip timing (Figure S5). However a portion of the strong signals identified preceding failure are likely from an ongoing event, which makes this analysis much less robust.

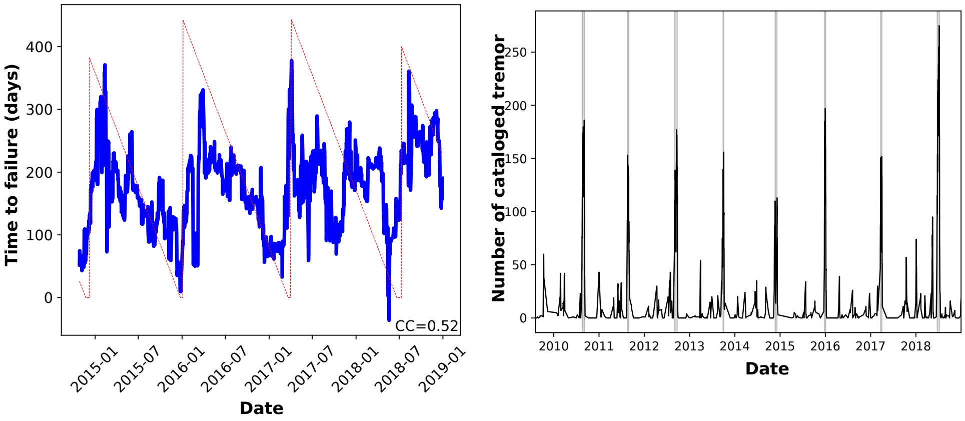

Supplementary Figure 6. We can also replicate the analysis by using bursts in the tremor catalog, localised around our area of interest, to identify failure times. Again, this has the disadvantage of being more spatially local, and introduces contamination from ongoing events. Moreover, the catalog only starts in late 2009 and leads us to discard about a third of the seismic data available (most seismic stations start recording in late 2005), which is detrimental to training ML models. Left: estimations of slip timings on the testing set, using a model trained bursts of cataloged tremor around Vancouver Islands. Right: identification of slip timings (gray shaded areas), from the rates of cataloged tremor smoothed over 10 days. The results use data from station B001, with a 3-month smoothing windows, and a 50%-50% contiguous train-test split.

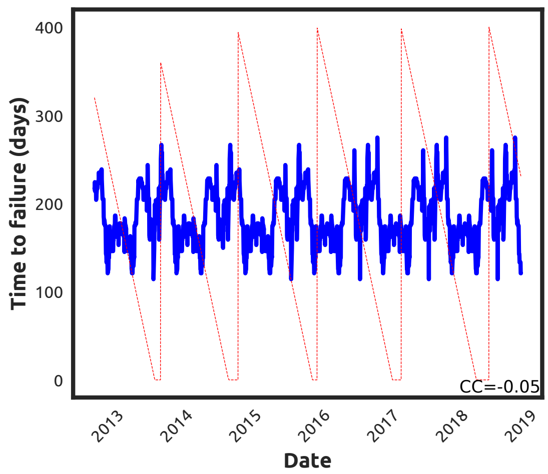

Supplementary Figure 7. Performance in testing when using a seasonal sinusoidal to estimate slip failure times. To demonstrate that our results rely on signals that are likely from tectonic origin and are not driven by seasonal phenomena, we perform a simple test. Instead of using our seismic features to estimate the timing of slow slip events, we rely on a simple yearly sinusoidal. We find that the algorithm is unable to map this seasonal sinusoidal to slip timings.

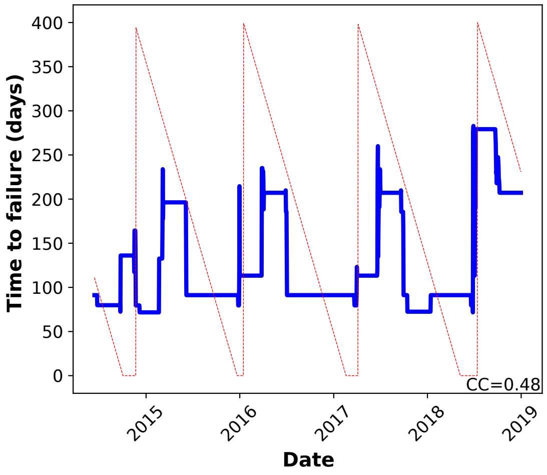

Supplementary Figure 8. Performance in testing when using known cataloged tremor to estimate slip failure times. Our results do not seem to be primarily driven by known cataloged tremor. The figure below shows the results of a similar analysis, limited to times when tremor is observed in the PNSN catalog.[1] We compute the same features, but instead of analysing continuous daily seismic waveforms, we only keep the portions of waveforms where tremor is present (and set the rest to zero). A model built on catalogued tremor alone does not perform nearly as well to estimate failure times. These results use data from station B001, with 3-month smoothing windows.

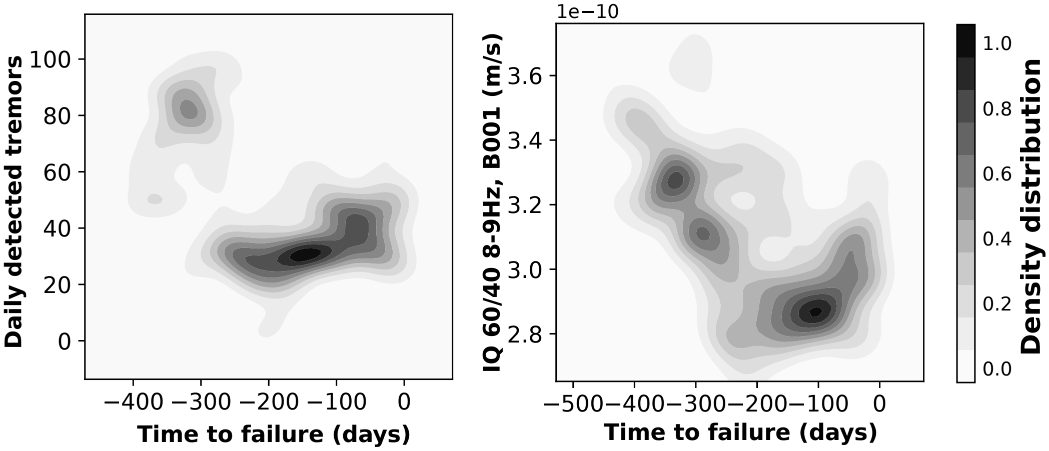

Supplementary Figure 9. Left: daily tremor detections made by **neural network** (see Supplementary Methods), smoothed over the same windows as our features (3 months). An increase in detected tremors becomes apparent approximately 100 days before failure. Right: evolution of seismic noise for comparison (same as in Figure 4 of the main text). Our main hypothesis is that the exponential build-up in seismic noise before failure is linked to slow slip nucleation, because it occurs within tremor frequency bands. The fact that tremor detections also increase around the same time is a strong indicator that our feature is indeed capturing an intensification in tremor activity, and is therefore reflects physical changes linked to the nucleation of the event. Most of these **tremors** are of small amplitude, and not included in existing catalogs, which explains why an analysis based on catalog tremor alone does not perform well.

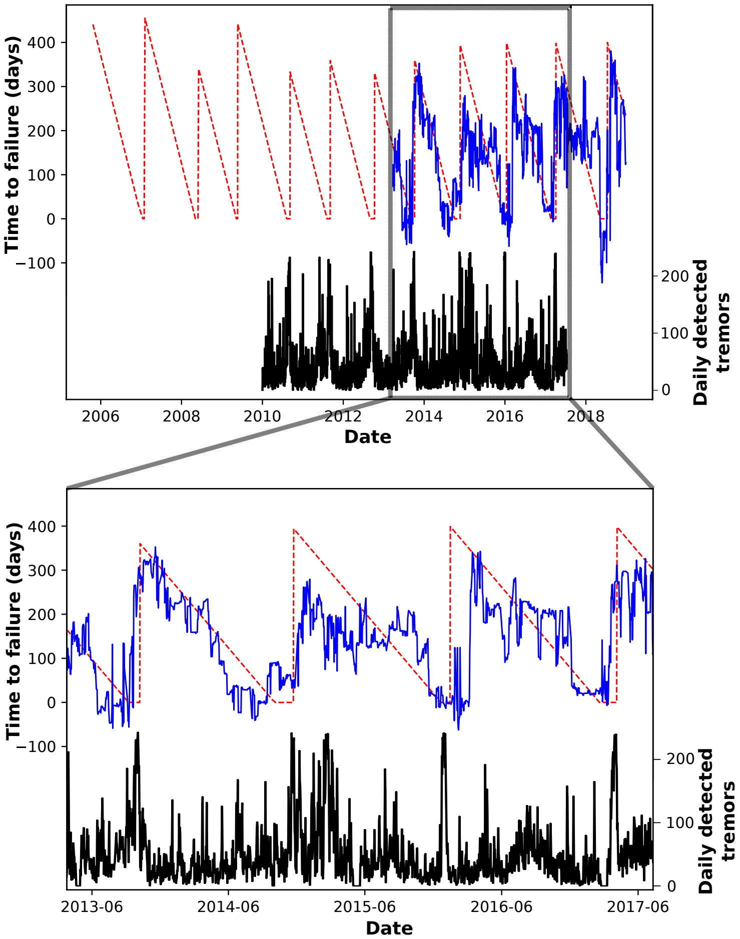

Supplementary Figure 10. Top: evolution of daily detected tremor over the entire period analyzed in our previous work (right axis), with comparison to ML estimates of time to failure (left axis). Bottom: zoom of the grey box area, where both tremor detections and ML estimates are available.

Supplementary Methods

Using GPS for slip timing identification.

The tremor logs, available on the PNSN website, are web pages that analyze each slow slip event, and provide a beginning and an end day for each event based upon the tremor detections made by the institution (starting in 2003). We chose to rely on these timings because they encompass the entire Cascadia area, and because data from the PNSN tremor catalog itself only starts in late 2009 – which would lead us to discard a third of the available data.

As another test for our approach, we use GPS displacement rates to identify failure timing (**Supplementary Figure 4**). We rely on GPS data from the Western Canada Deformation Array (WCDA), processed by the USGS Pacific Northwest Network.[2]

We use as ground truth times of failure obtained from GPS (Figure S4 B). A slow slip event corresponds to reverse motion of the GPS compared to the inter-SSE period (here, positive displacement rates are indicated in Figure S4 C). The time of failure corresponds to the time when GPS displacement rate becomes positive, in our reference. It is important to note that because the GPS data are noisy and must be smoothed to identify slip events, and because GPS stations are located at the surface potentially far from the fault region where the slow slip event actually begins, inferred times of failure are likely to differ from the actual timings of slow slip events. Therefore we consider GPS displacement rate as an imperfect proxy of slip timing on the fault interface. This is also why we chose to rely on the timings identified in the PNSN Tremor Log in the main text. In particular, the identified failures last longer than those defined by the PNSN tremor log because of the smoothing. The beginning of slow slip events seems to be relatively well captured, but

tends to occur after local bursts of tremor start (Figure S4 C).

We use the GPS station ALBH, which is located on Vancouver Island. This GPS station has been shown to be particularly sensitive to slow slip motion in the literature, and has been historically used to characterize slow slip.[3] We use **the horizontal displacement rates projected on the southwest direction**, such that a negative displacement rate corresponds to the loading phase of the cycle (in the North East direction), and a positive displacement rate corresponds to displacement towards the South-West (slow slip events). Identified **times of failure** from GPS are shown with gray vertical shading in the upper right portion of Figure S4 C, with local tremor rates from the PNSN catalog[1] for comparison.

We find that our analysis remains robust when we use GPS to identify the failure times; this is not surprising, as most of the events identified in the GPS are still characterized by approximately 14-month periodicity, matching the periodicity of our observed feature cycles. The results in Figure S4 A use the best seismic station (B001), and 3-month smoothing windows.

Detection of tremor with neural networks

In our recent work,[4] we built a neural network algorithm to identify tremor events on a single seismic station. This algorithm is able to detect many more tremor events compared to the catalog is was trained on. In particular, the fact that it detects tremor on a single station is likely to improve detection of low-amplitude **tremors** (which are difficult to identify using standard array-based techniques, as they do not appear on many seismic stations). **In Supplementary Figures 9 and 10, we use the detections made by our algorithm on station NLLB of the CN[5] network.** Note that the model detects whether a 5-min piece of waveform contains tremor, but does not specify how many **distinct occurrences of tremor** take place during these 5 minutes.

Therefore, the daily detections presented below are counts of 5-min time windows that include **tremor** according to our algorithm.

Supplementary References

[1] Wech, A. G. & Creager, K. C. Automated detection and location of Cascadia tremor. *Geophysical Research Letters* **35** (2008).

[2] Murray, J. R. & Svarc, J. Global positioning system data collection, processing, and analysis conducted by the u.s. geological survey earthquake hazards program. *Seismological Research Letters* **88**, 916 (2017).

[3] Rogers, G. & Dragert, H. Episodic tremor and slip on the cascadia subduction zone: The chatter of silent slip. *Science* **300**, 1942–1943 (2003).

[4] Rouet-Leduc, B., Hulbert, C., McBrearty, I. W. & Johnson, P. A. Probing slow earthquakes with deep learning. *Geophysical Research Letters* **47**, e2019GL085870 (2020).

[5] Canadian national seismograph network. *Geological Survey of Canada* (1989).
